# Enhancing human-human musical interaction through kinesthetic haptic feedback using wearable exoskeletons: theoretical foundations, validation scenarios, and limitations

**DOI:** 10.3389/fpsyg.2024.1327992

**Published:** 2024-03-07

**Authors:** Aleksandra Michałko, Nicola Di Stefano, Adriaan Campo, Marc Leman

**Affiliations:** ^1^Faculty of Arts and Philosophy, IPEM Institute of Psychoacoustics and Electronic Music, Ghent University, Ghent, Belgium; ^2^Institute of Cognitive Sciences and Technologies, National Research Council of Italy (CNR), Rome, Italy

**Keywords:** instrumental music training, haptic feedback, collaborative learning, technology mediation, exoskeletons, wearable robotics, human-human interaction (HHI), kinesthetic feedback

## Abstract

In this perspective paper, we explore the use of haptic feedback to enhance human-human interaction during musical tasks. We start by providing an overview of the theoretical foundation that underpins our approach, which is rooted in the embodied music cognition framework, and by briefly presenting the concepts of action-perception loop, sensorimotor coupling and entrainment. Thereafter, we focus on the role of haptic information in music playing and we discuss the use of wearable technologies, namely lightweight exoskeletons, for the exchange of haptic information between humans. We present two experimental scenarios in which the effectiveness of this technology for enhancing musical interaction and learning might be validated. Finally, we briefly discuss some of the theoretical and pedagogical implications of the use of technologies for haptic communication in musical contexts, while also addressing the potential barriers to the widespread adoption of exoskeletons in such contexts.

## 1 Introduction

One of the primary motivations for human collaborative interaction is the pursuit of reaching goals that typically go beyond the scope of individual capabilities (Jarrassé et al., 2012; Sebanz and Knoblich, 2021). Such forms of collaboration commonly rely on the exchange of verbal and sensory information among the interacting humans. For instance, a table, too heavy for one person, can be moved collaboratively by two individuals. Effective collaboration can reduce cost, time and overall effort required for the task.

In this paper, we consider the question of how collaborative interactions might be enhanced through the use of technology with the twofold aim of increasing effectiveness and reducing the effort required from humans. We believe that playing music is a suitable context in which to test this hypothesis, for at least two reasons. First, because playing music is paradigmatic for human-human collaboration and sensorimotor interaction (D’Ausilio et al., 2012, 2015; Nijs, 2019). Second, because providing a measurable definition of the augmented level of collaboration in the domain of music is seemingly facilitated by the fact that several quantifiable sensorimotor parameters are at stake in music interaction (Biasutti et al., 2013; Volpe et al., 2016; Campo et al., 2023). For example, a stable musical rhythm and tempo require each musician in an ensemble to coordinate their actions in response to the rhythms played by other musicians. The co-regulation of the ensemble can be measured by onset extraction and subsequent analysis of timing intervals (for example, by using trackers of periods based on Kalman filtering, Leman, 2021).

Over the last decades, musical interactions have been conceptualized within the broad theoretical framework of embodied cognition, according to which human action and body movement play a central role in the experience of music, among others sensorimotor activities (Leman, 2007; Biasutti et al., 2013). The question we want to address here deals with the possibility of technologies to enhance these embodied interactions. We envision the possibility that enhancing the exchange of information among users by providing an additional channel of information based on haptic feedback might help boost the collaborative dimension of human-human interaction. The challenge is whether these technologies can enhance collaborative interactions and how to empirically demonstrate their positive effects, if any.

To address these questions, we first outline the theoretical foundation on which our explorations are based, namely the embodied music cognition framework, focusing especially on the concepts of action-perception loop and entrainment (Section 2). Thereafter, we examine the nature of haptic information (Section 3) and the role of haptic exoskeletons to communicate such information (Section 4). We present two experimental scenarios that could demonstrate the effectiveness of the technology for enhancing musical interaction (Section 5). Finally, we discuss the theoretical and pedagogical implications of this perspective technology as well as its limitations (Section 6).

## 2 Action-perception cycles and entrainment

Using technology to add sensory feedback providing information on other musicians could be seen as a natural extension of the human action-perception cycle, in which external auditory feedback generates a motor output, which in turn produces auditory feedback and another motor response (Kaspar et al., 2014; Leman and Maes, 2014; Pinardi et al., 2023). The technological augmentation of the sensory feedback with the addition of haptic information would influence action-perception cycles via entrainment, which can be conceived of as a dynamic adaptation of the actions of a subject influenced by the actions of another subject (Kaspar et al., 2014; König et al., 2016; Pinardi et al., 2023). Both action-perception loops and entrainment are fundamental concepts that underpin the theoretical foundation for technology-mediated collaborative feedback (Kaspar et al., 2014).

Music performance is an excellent domain for exploring this kind of feedback as it involves a tight coupling of perception and action. The idea that music taps into a shared action repertoire for both the encoding (playing) and decoding (listening, dancing) of music has been central to the embodied music cognition framework (Leman, 2007; Barsalou, 2008; Godøy and Leman, 2010; Leman and Maes, 2014; Schiavio, 2014). It connects with recent trends in cognitive science for understanding human action and experience (Prinz, 1997; Sheets-Johnstone, 1999; Thelen, 2000; Gibbs, 2006; Shapiro, 2011; Witt, 2011). The conceptual framework generated a solid body of empirical research that provided evidence for the role of human motor system and body movements in music perception, showing that how we move affects how we interpret and perceive rhythm, and what we hear depends on how we move and *vice-versa* (Phillips-Silver and Trainor, 2005, 2007; Maes and Leman, 2013; Manning and Schutz, 2013; Maes et al., 2014a). For instance, Maes et al. (2014b) showed that music-driven gestures could facilitate music perception and Moura et al. (2023) recently showed that the tonal complexity of music tightly couples to knee bending movements of saxophone players performing the music.

In embodied music cognition, sensorimotor coupling is seen as a lower-level form of action-perception coupling. Sensorimotor coupling covers multiple proprioceptive and exteroceptive cycles at work during playing, including haptic feedback (Leman, 2007). Consider a violinist who receives proprioceptive feedback from their own musculoskeletal system holding the violin, as well as acoustic feedback from the sound produced through air and bone conduction. At higher-level, one could mention gestures, body movement and actions, connected to perceived structures, for example. In this embodied perspective, the collaborative interaction with co-regulated actions in view of shared musical goal is one of the most characterizing aspects of any ensemble, from duet, to trio, to big orchestra (Biasutti et al., 2013; Glowinski et al., 2013; Badino et al., 2014). The action-perception cycle can be seen as the ability in each subject to react coherently to changes that occur in the ensemble (Biasutti et al., 2013). Last but not least, action-perception cycles exist also between the players and the listeners, with feedback coming through multiple sensory channels, such as the visual channel (facial expressions, gaze, gestures and movements), the acoustic channel (e.g., singing, hand clapping), the olfactory channel (e.g., different smells).

The action-perception cycle can thus be seen as the engine driving co-regulated actions at group level. Entrainment has been suggested to be a useful concept (Clayton, 2012; Keller et al., 2014; MacRitchie et al., 2017; Clayton et al., 2020) to capture the dynamic change in musical actions. Clayton (2012) distinguished between three different levels of the entrainment, namely intra-individual, inter-individual, and inter-group entrainment. Intra-individual entrainment refers to processes that occur within a particular human being, such as the entrainment of networks of neuronal oscillators or the coordination between individual body parts (e.g., the limbs of a drummer). Inter-individual (or intra-group) entrainment concerns the co-ordination between the actions of individuals in a group, as might occur chamber or jazz ensembles. Finally, inter-group entrainment concerns the coordination between different groups.

Notably, the interaction and synchronization between individuals in Western group ensembles primarily occurs through visual and auditory channels (Goodman, 2002; Biasutti et al., 2013). This is because the nature of the task, where musicians play individual instruments, prevents the exchange of information between players via physical touch. What is at stake in this paper, however, is the emergence of novel technologies that could facilitate the haptic communication among musicians, thereby fostering a more profound sensorimotor interaction between them.

## 3 Haptic feedback

Having defined the basic concepts for collaborative interaction, it is of interest to consider how haptic feedback affects music playing. In general, haptics refers to the study of how humans perceive and manipulate touch, specifically through kinesthetic (force/position) and cutaneous (tactile) receptors (Hannaford and Okamura, 2016). Research has demonstrated the significance of touch and haptic information in facilitating human interaction, such as dancers synchronizing their movements through touch (Sofianidis and Hatzitaki, 2015; Chauvigné et al., 2018) or children learning to walk with parental assistance (Berger et al., 2014). Moreover, haptic feedback might be used to provide visuo-spatial information to people with visual impairments or blindness (Sorgini et al., 2018, for a review). For instance, a recent work by Marichal et al. (2023) a system for training basic mathematical skills of children with visual impairments based on the combination of additional haptic and auditory information.

In music learning, haptic information serves as a powerful tool, enabling learners to adjust their movements and develop new musical actions (Abrahamson et al., 2016). Teachers use touch to physically guide learners’ movement, to direct their attention to their bodies and to receive haptic information about students’ bodies, such as tension, which helps them guide, assess, and adapt their touch to the learners’ needs (Zorzal and Lorenzo, 2019; Bremmer and Nijs, 2020). Remarkably, haptic communication in music education depends on music teachers’ understanding of the ethical boundaries of physical contact with their learners, as learners might be highly sensitive to being touched (Bremmer and Nijs, 2020).

**Figure 1:**
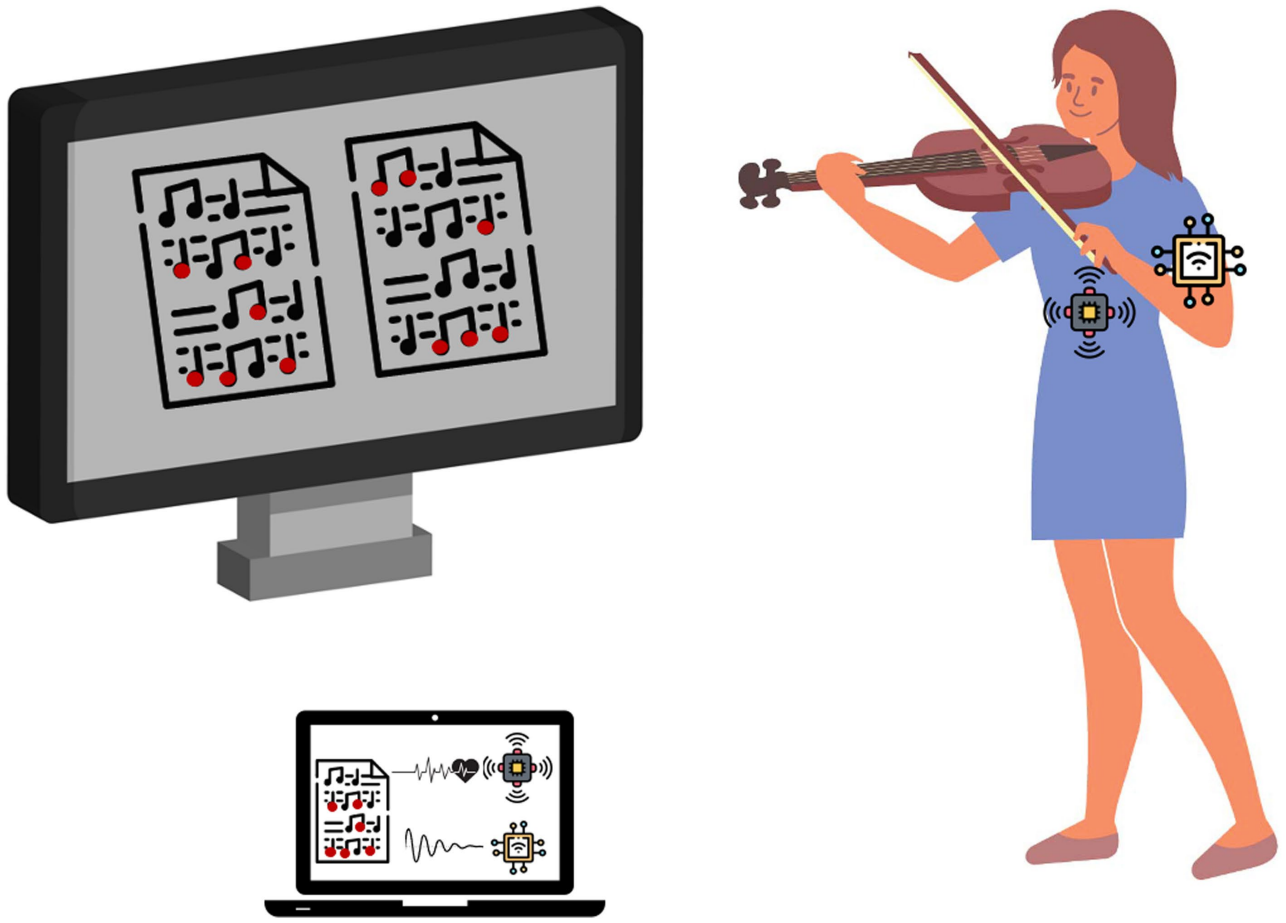
A conceptual representation of state-of-the-art technologies developed for music playing. The systems typically include multiple sensors that track students’ physiological processes and provide real-time feedback on various movement and audio parameters related to their performance. They indicate when a player deviates from the target movement trajectory, adopts incorrect body posture, or deviates from the target pitch. They are usually designed to be used individually.

Over the last decade, several haptic feedback-based devices for instrumental music training have been developed. Most of them are based on vibrotactile stimulators that provide real-time feedback whenever the player deviates from a target movement trajectory (van der Linden et al., 2011), has incorrect body posture (Dalgleish and Spencer, 2014), deviates from the target pitch (Yoo and Choi, 2017), or provide guidelines to accurately execute rhythmic patterns that require multi-limb coordination (Holland et al., 2010) (see Figure 1 for the conceptual representation of these devices). Nevertheless, validation studies of these devices show that the efficiency of vibrotactile feedback is dependent on the player’s attentional needs, with some individuals experiencing difficulties concentrating on playing due to frequent and/or unclear vibrotactile input (van der Linden et al., 2011; Yoo and Choi, 2017). These findings suggest that vibrotactile feedback may not be the most effective type of haptic feedback for facilitating sensorimotor skill acquisition (van Breda et al., 2017).

Surprising as it may seem, however, little research has been conducted on technologies that actively assist musicians in developing prediction schemes while playing via physical forces that guide sensorimotor control (e.g., when the teacher holds and guides the student’s arm while playing). Yet, kinesthetic haptic feedback, which transmits force and position information of the motor target movement, is considered a more promising type of haptic feedback for promoting the development of sensorimotor abilities as it enables fine-grained movement guidance in space and time (Fujii et al., 2015; Pinardi et al., 2023). For example, the validation study of the Haptic Guidance System apparatus (HAGUS), a kinesthetic device that targets the optimal rendition of wrist movements during drumming, suggests that force haptic guidance is significantly more effective than auditory guidance alone at communicating velocity information (Grindlay, 2008).

Furthermore, with the possible exception of the MoveMe system (Fujii et al., 2015), which connects an expert and a beginner via two haptic robots to enable the expert to guide and correct the beginner’s hand movement in real time, it seems that none of the previously mentioned devices tackle interpersonal interaction, which is vital in joint music performance and learning (D’Ausilio et al., 2015; Nijs, 2019).

## 4 Haptic exoskeleton technology

Exoskeletons are body-grounded kinesthetic devices designed to closely interact with the structures of the human body. They are positioned on the user’s body to provide sensory touch information, serving as amplifiers to enhance, strengthen, or recover human locomotor performance (Zhang et al., 2017). In this paper, we focus on lightweight, portable upper-limb exoskeletons with spring mechanisms, consisting of two haptic robotic modules for the shoulder and elbow. In addition to shoulder and elbow active modules, the robotic interface also includes passive elements that stabilize the device and evenly distribute the reaction forces over the user’s body, as well as torque-controlled, compliant actuators for accurate haptic rendering. This design allows the user to move the shoulder and elbow freely while receiving assistive action for flexion-extension.

This system is suitable for instrumental music training, particularly for these instruments that require relatively large arm movements for sound production, like strings or percussion instruments (see, e.g., the CONBOTS project, conbots.eu). Such a robotic system could convey force and position information on the motor target to the user, potentially improving prediction accuracy, accelerating, and facilitating the learning process. Exoskeletons might have the capability to not only provide haptic information to the user and guide his/her movement based on a targeted trajectory, but also to enable haptic communication between two exoskeleton-wearing users, allowing them to obtain accurate information on each other’s movements and forces in real-time (Takagi et al., 2017, 2018). The physical communication between users could be thus established by physically connecting two exoskeleton-wearing humans via a coupling mechanical impedance, which is haptically rendered in correspondence with the exoskeleton’s attachment points. As a result, users should experience a virtual impedance in accordance with their joints, whose equilibrium positions are based on the joint positions of the other user. Notably, the system’s visco-elastic properties enable real-time modulation of virtual impedance during tasks by adjusting spring/damping coefficients.

## 5 Music interaction scenarios

**Figure 2:**
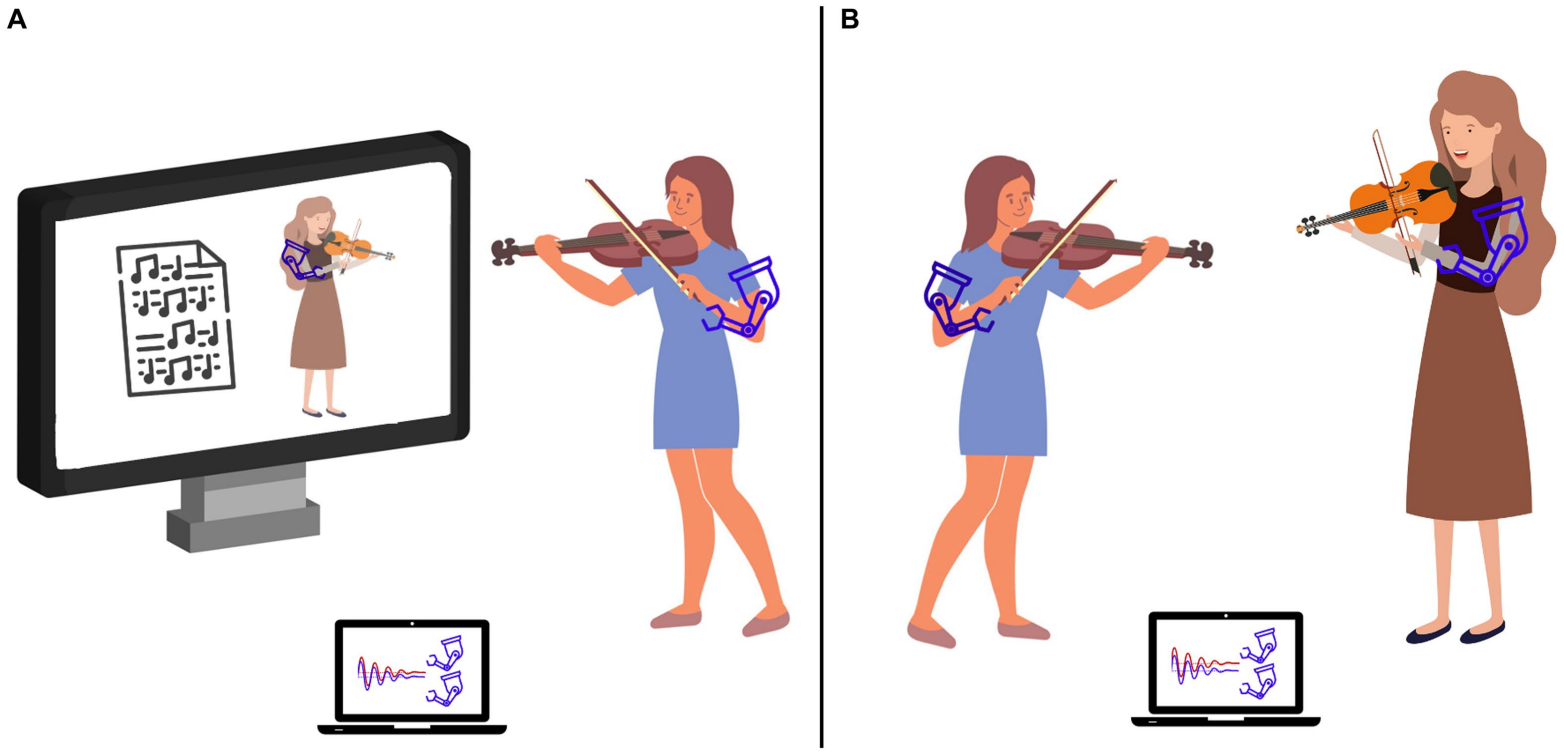
A conceptual representation of the real-time kinesthetic haptic feedback mediated by exoskeletons to enhance learning and performance. Besides providing haptic information to the users and guide their movement based on a targeted trajectory **(A)**, this technology has the capability to enable the exchange of forces between two exoskeleton-wearing users, allowing them to obtain haptic information on each other’s movements in real-time thus enhancing sensorimotor interaction and facilitating entrainment effects between users without interfering with the general performance and flow of the lesson or rehearsal **(B)**.

In this section, we propose two experimental scenarios in which to test the efficacy of real-time haptic feedback mediated by exoskeletons for musical tasks. The first scenario is based on the unidirectional human-machine interaction (see Figure 2A). The second scenario is based on the bidirectional human-human interaction (see Figure 2B).

### 5.1 Unidirectional human-machine interaction

The first scenario is based on a music playback system for violin beginners which involves a student who plays along with a video showing the teacher. The video is augmented with haptic exoskeletons providing information about the teacher’s movement. Wearing the lightweight exoskeleton, the student receives both auditory, visual, and haptic information while performing the task (see Figure 2A). The student’s goal is to synchronize their bowing movements with the ones of the teacher. Besides following the teacher’s instructions audio-visually, students can also benefit from the haptic information on the actual movements performed during the lesson, thus introducing an additional information channel. It would be reasonable to hypothesize that such additional haptic feedback can enhance the action-perception cycles by providing additional information which fuels the student’s sensorimotor loop. The enrichment of action-perception cycles might have, in turn, cascading positive effects on student’s performance of bowing movements and on learning pace. Improvements in bowing can be quantitatively assessed by comparing the parameters of movements (e.g., trajectory and smoothness) of a group of students who train with the haptic teacher with a control group of learners training with the video playback only.

### 5.2 Bidirectional human-human interaction

The second scenario shifts from individual training to joint music performance, where individual musicians operate as processing units within a complex dynamical system, with the objective to drive each other toward a common esthetic goal (D’Ausilio et al., 2012). For instance, string musicians in an orchestra who need to possess precise gesture coordination and motion synchronization to produce a unified, harmonious, and cohesive section sound. Performers currently exchange sensorimotor information through visual and auditory feedback (Biasutti et al., 2013). The central question at hand is whether bidirectional haptic feedback can serve as additional feedback that improves violinists’ co-regulated action in terms of bowing gestures coordination, motion synchronicity, tempo stability, volume balance, and tone blending.

In order to test it in an experimental setting, pairs of violinists (with varying expertise levels) might be asked to perform the same piece multiple times in four different conditions: audio-visual-haptic, audio-visual, audio-haptic, and audio-only condition. The experimental task would be to perform a musical piece as well as possible as a group, especially in terms of tempo changes such as ritardando and accelerando, which require precise rhythmic co-regulation and synchronization. In order to exclude the influence of visual information in the audio-haptic and audio-only conditions, participants could be separated by a black curtain. The bidirectional haptic feedback would be activated through exoskeletons worn by participants throughout the experiment in the audio-visual-haptic and audio-haptic conditions only. Importantly, violinists should not be informed that the forces they feel are coming from their colleague. We hypothesize that the presence of haptic feedback would improve violinists’ performance because it provides direct or non-mediated sensorimotor feedback on motor parameters of bowing gestures. In contrast, indirect (or mediated) sensorimotor feedback via auditory and/or visual channels requires the translation from one modality (audio, visual) to another (motor). Our hypothesis is that direct (non-mediated) feedback will prove more effective than indirect (mediated) feedback. This improvement is expected to be even more significant in violinist pairs with less experience.

## 6 Discussion

### 6.1 Extension of the theoretical basis

As described in Section 2, the theoretical underpinning of collaborative interaction relies on the concepts of action-perception cycle and entrainment. Obviously, both concepts need further elaboration and refinement, for example in the direction of predictive processing (Clark, 2013), and expressive information exchange (Leman, 2016). The success of integrating haptic exoskeleton technology in a natural action-perception cycle indeed draws upon the ability of the user to feed and form predictive models of acting and interacting. The concept of entrainment thereby offers a dynamic perspective for understanding co-regulated actions. Concurrently, enabling bidirectional haptic feedback between two musicians could contribute to the emergence of novel research paradigms, the development of new metrics, and a deeper understanding of the mechanisms that govern action-perception cycles and entrainment.

To date, the level of unidirectional interaction can be assessed through the metrics developed in Campo et al. (2023), which involves a comparison between movement information captured via a motion capture system with infra-red cameras, based on kinematic features, such as movement smoothness (SPARC, see Balasubramanian et al., 2015) or similarity of the bow gestures of the student relative to the teacher. Additional metrics can rely on angular information of joints provided by the exoskeleton. The bidirectional interaction could be measured by entrainment as it aims at indicating the effect of collaborative interaction via the dynamic change of actions. The angular velocity of the shoulder and elbow of each violinist using the exoskeleton, as well as the movement displacement of the upper and lower arm using a motion caption system can be used as signals. Bi-directional interaction may then be mapped out using techniques from dynamical systems analysis, such as recurrence analysis (Demos et al., 2018; Rosso et al., 2023), or cross-wavelet transforms (Torrence and Compo, 1998; Eerola et al., 2018).

### 6.2 Extension of the scenarios

Additional experimental scenarios in collaborative haptic interaction will be needed to allow for a clearer understanding of the effects of kinesthetic haptic feedback. Of particular interest might be the extension of the music playback scenario from unidirectional to bidirectional interaction. Differently from the bidirectional human-human scenario, in the playback scenario the bidirectional exchange would occur between the user and an AI teacher, trained via machine learning of bowing movements. Of course, the development of a bidirectional platform for interaction is a challenging task involving human-based AI. The unidirectional approach as well as understanding of adaptive mechanisms that might coregulate dynamics of haptic human-human interactions are steps toward such a bidirectional system equipped with advanced machine intelligence for interacting.

Furthermore, understanding the needs, preferences, and concerns of users is essential for the technology’s successful adoption and further development (Bauer, 2014; Mroziak and Bowman, 2016; Michałko et al., 2022). Since musicians typically rely on auditory and visual information for communication (Goodman, 2002; Biasutti et al., 2013), they may initially find this additional communication channel as distracting rather than beneficial. Therefore, to achieve successful technology adoption, a participatory design should be carefully implemented, encompassing various validation scenarios and intense interaction between the developers and end users at every stage of technology development (Bobbe et al., 2021; Michałko et al., 2022). This approach will not only help shape the design of the technology to better meet users’ expectations (Bauer, 2014; Mroziak and Bowman, 2016), but it can also help verify in a proactive way the technical effectiveness of the devices themselves.

### 6.3 Extending music education

Traditionally, learning to play a musical instrument is mediated by verbal and non-verbal communication between the teacher and the trainee, including hand gestures, physical guidance, apart from the use of verbal imagery and metaphors (Williamon and Davidson, 2002; Nijs, 2019; Bremmer and Nijs, 2020). Each of these communication channels has been demonstrated to play a positive role in transferring instrumental music instruction and ideas (Simones et al., 2017; Meissner and Timmers, 2019; Bremmer and Nijs, 2020), but their drawbacks have also been identified, such as ambiguous interpretation, delayed feedback, and the fact that they only provide a rough approximation of the target movement (Hoppe et al., 2006; Howard et al., 2007; Grindlay, 2008). Some of the currently available devices provide real-time objective feedback to overcome these limitations (Schoonderwaldt and Demoucron, 2009; Blanco et al., 2021), but they focus on movement and posture monitoring, reinforcing the master-apprentice approach, which has been criticized for emphasizing reproductive imitation over creative music making (Nijs, 2019; Schiavio et al., 2020). Exoskeletons may be a promising alternative to current technologies because they enable real-time, direct sensorimotor feedback between two users thereby supporting teacher-student or peer interactions, which are key to students’ long-term music engagement (Davidson et al., 1996; Zdzinski, 2021). Exoskeleton technology might also enhance learning and current communication channels (auditory and visual) without interfering with lesson performance or flow (see Figure 2B). It could be of interest to investigate the impact of various haptic modes of exoskeletons (bi-directional vs. unidirectional) on different pedagogical models, as well as its impact on interpersonal relationships between teacher-student and peers.

### 6.4 Extension to other domains

Haptic devices have been developed in the field of rehabilitation (Wu and Chen, 2023), sport science (Spelmezan et al., 2009), and mixed reality and gaming industry (Moon et al., 2023). However, little research has pointed out the potential benefits of collaborative interaction with force haptic feedback through exoskeletons. So far, research has predominantly concentrated on the action-perception cycle rather than the entrainment dynamics typical of collaborative interactions. Lightweight exoskeleton technology offers a huge potential in several (non-musical) domains of application where bidirectional, i.e., collaborative, interaction is useful, for example, in physiotherapy where movements are guided in interaction with the therapist (Bjorbækmo and Mengshoel, 2016). It could be of interest to investigate whether incorporating bidirectional haptic feedback via exoskeletons could improve the relationship between physiotherapists and patients, thereby allowing for a faster recovery process.

### 6.5 Limitations

The current state-of-the-art exoskeletons are considered lightweight compared to, for instance, exoskeletons used in factories; however, they might still be perceived as bulky and heavy especially when worn by children. The scalability of anthropomorphic features is also a critical issue for exchanging meaningful feedback between, say, a child and an adult, which are often paired in training/learning contexts. These exoskeletons therefore require the assistance and control of experts for correct attachment to limbs and for setting the different interaction paradigms such as unidirectional and bidirectional. More effort will be required for making these devices more user friendly and ready to be used out of the laboratory. Another important factor to investigate is the potential time delay between two users when exchanging forces. Further limitations are concerned with the restricted domain of application in music learning, with arm exoskeletons being less useful when playing brass or woodwind instruments, which produce sound through air emission and finger movements rather than shoulder and elbow movements.

## 7 Conclusion

In this paper, we envisioned the possibility of using lightweight robotic exoskeletons to allow for the exchange of haptic information during musical interactions. We introduced two possible validation scenarios involving violin playing to explore and test the feasibility of adding haptic information to the audio and visual channels on which collaborative interaction is based. These scenarios leverage on music playback systems and real-time interactive music making, especially in the context of learning. However, the application of kinesthetic haptic systems that allow for bidirectional force exchange may potentially extend to various domains, encompassing physical rehabilitation, sports, gaming, and those fields in which gestures and limb movements benefit from haptic guidance. In such scenarios, exoskeletons can be employed to enhance the entrainment effect, ultimately fostering improved co-regulation activities.

## Data availability statement

The original contributions presented in the study are included in the article/supplementary material, further inquiries can be directed to the corresponding author.

## Author contributions

AM: Writing – review & editing, Writing – original draft, Visualization, Conceptualization. NDS: Writing – review & editing, Writing – original draft, Supervision, Conceptualization. AC: Writing – review & editing. ML: Writing – review & editing, Writing – original draft, Supervision, Funding acquisition, Conceptualization.
